# Medium Bandgap D-A Type Photovoltaic Polymers Based on an Asymmetric Dithienopyran Donor and a Benzotriazole Acceptor

**DOI:** 10.3390/polym9100516

**Published:** 2017-10-17

**Authors:** Junyi Hu, Xiaochen Wang, Fan Chen, Bo Xiao, Ailing Tang, Erjun Zhou

**Affiliations:** 1 CAS Key Laboratory of Nanosystem and Hierarchical Fabrication, CAS Center for Excellence in Nanoscience, National Center for Nanoscience and Technology, Beijing 100190, China; hujy@nanoctr.cn (J.H.); chenf@nanoctr.cn (F.C.); xiaob@nanoctr.cn (B.X.); tangal@nanoctr.cn (A.T.); 2 University of Chinese Academy of Sciences, Beijing 100049, China

**Keywords:** polymer solar cells, asymmetrical building blocks, dithienopyran (**DTPa**), benzotriazole (**BTA**)

## Abstract

Conjugated polymers based on the donor of an asymmetric 5*H*-dithieno[3,2-b:2′,3′-d]pyran (**DTPa**) and the acceptors of benzo[d][1,2,3]triazole (**BTA**) or di-fluorinated benzo[d][1,2,3]triazole (**ffBTA**) with thiophene as π-bridge were designed and synthesized. Two asymmetric-building-block-containing polymers (ABC-polymers) possess a strong and broad absorption in the range of 300–750 nm and medium optical bandgap of 1.73 and 1.77 eV for **PDTPa-TBTA** and **PDTPa-TffBTA**, respectively. Polymer solar cells using **PDTPa-TBTA** as donor and [6,6]-phenyl-C_71_-butyric acid methyl ester (PC_71_BM) as an acceptor exhibited power conversion efficiencies (PCE) of 2.22% with a *V*_oc_ of 0.58 V, a *J*_sc_ of 6.04 mA/cm^2^, and an FF of 63.41%. The introduction of fluorine substituents on the **BTA** unit evidently influenced the optical and photovoltaic properties. Interestingly, although the HOMO energy level indeed decreased, **PDTPa-TffBTA** showed a decreased *V*_oc_ of 0.52 V in solar cells. Combined with an obviously enhanced *J*_sc_ of 10.23 mA/cm^2^, and an outstanding FF of 0.64, the PCE of solar cells based on **PDTPa-TffBTA** was improved by nearly 55%, reached 3.43%. Our results indicate that the **BTA** unit can be used to construct ABC polymers with a medium bandgap, and the introduction of fluorine on the **BTA** unit is also effective in improving the photovoltaic performance.

## 1. Introduction

Over the past few decades, polymer solar cells (PSCs), given the advantages of a low cost, a light weight, and flexibility, have attracted extensive attention for the generation of affordable, clean, and renewable energy [1]. Generally, efficient PSCs adopt a bulk heterojunction device structure, utilizing a p-type conjugated polymer as a donor and an n-type material (fullerene derivatives, conjugated polymers, or small molecules) as an acceptor [2,3,4,5,6,7,8,9]. Traditionally, from the knowledge of some classic donor polymer system, such as poly(3-hexylthiophene-2,5-diyl) (P3HT) [10,11,12,13], people have believed that photovoltaic polymers with great promise should be region-regular, so symmetric building blocks have played a dominant role in the design and synthesis of D–A (donor–acceptor) type polymer photovoltaic materials. However, some effective asymmetrical building blocks have developed gradually to construct promising photovoltaic polymers, such as poly [[4,8-bis-[(2-ethylhexyl)oxy]benzo[1,2-b:4,5-b′]dithiophene-2,6-diyl][3-fluoro-2-[(2-ethylhexyl)carbonyl]thieno[3,4-*b*]thiophene-4,6-diyl]] (PTB7) and its analogues, with the best power conversion efficiency over 10% [14,15,16]. This made people realize that the lack of enough attention restricted the development of asymmetric-building-block-containing polymers (ABC polymers) [17]. Consequently, it has become very important to develop new asymmetrical building blocks and explore the relationship between photovoltaic performance and structures of ABC polymers. 

**Scheme 1:**
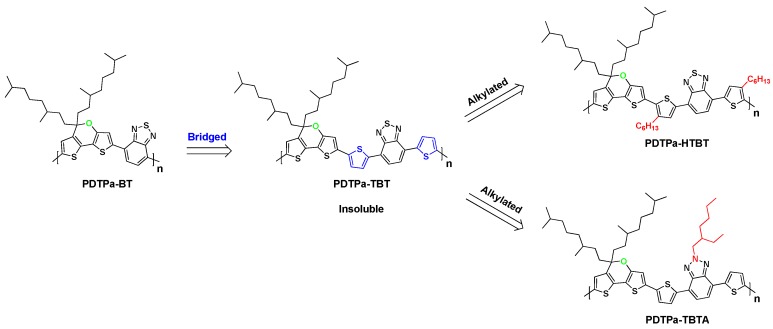
The chemical structures of the polymers based on **DTPa**, **BT**, and **BTA**.

On the other hand, in our previous research, we found that the characters of copolymers such as the frontier molecular orbital, the architecture of the molecule, and the optoelectronic properties could be significantly influenced by the π-bridge units. However, there is still an extreme lack of research on the effect of π-bridges on the properties of ABC polymers. Recently, we designed and synthesized conjugated polymers based on asymmetric dithieno[3,2-b:2′,3′-d]pyran (**DTPa**) donor and benzothiadiazole (**BT**) acceptors and shown in Scheme 1 [17,18]. When inserting a thiophene bridge between these two segments, the forming polymers suffer from poor solubility in common organic solvents. Therefore, we had to introduce hexyl substituents in the thiophene bridge to guarantee the solubility of the polymers for preparation and purification, as well as exploration of the properties of the polymer semiconductors in solution-processable photovoltaic devices. However, steric hindrance of the alkyl substituents led to an obvious increase in dihedral angles between the thiophene bridge and the **DTPa** unit and consequently affected the conjugation of the resulting polymers.

**Scheme 2:**
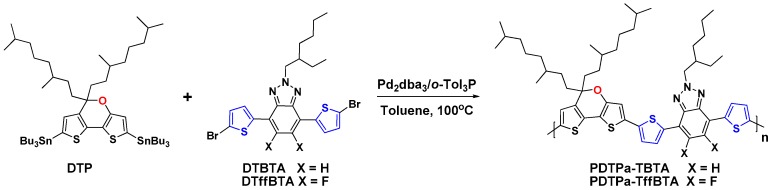
Chemical structures and synthetic routes of **PDTPa-TBTA** and **PDTPa-TffBTA**.

In order to solve this problem, benzo[d][1,2,3]triazole (**BTA**) was used instead of the **BT** acceptor. Among large amounts of electron-deficient building blocks, benzotriazole is one of the most widely used and excellent acceptors. Besides p-type polymers, **BTA** is also effective in constructing small non-fullerene molecules [19,20]. Compared to the analogue benzothiadiazole derivatives (**BTs**), **BTA**-based polymers provide the prominent advantage of facile incorporation of solubilizing alkyl chains onto the acceptor unit, rather than on the thiophene π-bridge. Therefore, placing the alkyl chain away from the polymer backbone on the **BTA** unit allows the polymer backbone to adopt a more planar conformation [21]. Thus, **BTA**-based D-A type polymers, combined with the electron-donating group of benzodithiophene [22,23,24,25,26,27], dithienylsilole [28], fluorene, phenylene, and carbazole [29], have been investigated in the past few years. However, **BTA** has not been copolymerized with asymmetric electron-donating building blocks. Therefore, in this work, we designed and synthesized D-π-A conjugated polymers combining asymmetric **DTPa** donor and **BTA** or **BTA** fluorinate acceptors, bridged by thiophene, to explore the effect of the symmetry and fluorination on the performance of photovoltaic polymers. The structures and synthetic routes of the polymers are shown in Scheme 2.

## 2. Experimental Section

### 2.1. Measurements and Characterization

The molecular weights of the polymers were measured via the gel permeation chromatography (GPC) method. The number-average molecular weights (*M*_n_), weight-average molecular weights (*M*_w_), and polydispersity index (PDI, *M*_w_/*M*_n_) were measured on a PL-220 (Polymer Laboratories Ltd., Shropshire, UK) chromatography connected to a differential refractometer with polystyrene as a reference standard and 1,2,4-trichlorobenzene as an eluent (at 150 °C). Elemental analyses were performed on a Flash EA 1112 analyzer (Thermo Electron Corporation, Waltham, MA, USA). Absorption spectra measurements of the polymers were carried out in a chloroform solution and films at room temperature, recorded on a Hitachi UH5300 spectrophotometer (Hitachi High-Technologies Corporation, Tokyo, Japan). The electrochemical cyclic voltammetry was performed on a Shanghai Chenhua CHI620 electrochemical workstation, with a platinum plate, an Ag/AgCl electrode, and a platinum wire as the working electrode, the reference electrode, and the counter electrode, respectively, in a 0.1 mol/L tetrabutyl ammonium hexafluorophosphate (Bu_4_NPF_6_) acetonitrile solution. Polymer thin films were formed by drop-casting of polymer solutions in CHCl_3_ on the working electrode and then dried in air. 

### 2.2. Fabrication of the Photovoltaic Devices

Organic solar cells were fabricated with the typical sandwich structure of ITO/PEDOT:PSS/active layer/Ca/Al. The tin-doped indium oxide (ITO) glass substrate as a positive electrode, Ca/Al metal electrode was evaporated as a cathelectrode, and the blend film of the polymer/PC_71_BM located as an active layer. Glass substrates coated with patterned ITO were cleaned by ultrasonication in detergent, water, acetone, and 2-propanol. Then, under a stream of nitrogen and subjected to the treatment of UV-Ozone for 15 min after drying. And coated by a thin layer of poly(3,4-ethylene dioxythiophene):poly(styrenesulfonate) (PEDOT:PSS), which was spin-coated from a PEDOT:PSS aqueous solution on the ITO substrate at a speed of 3000 rpm/s and annealed at 150 °C for 15 min in air atmosphere. The photosensitive layer was prepared by spin-coating a blend solution of polymer and PC_71_BM (1:1.5 or 1:2, by weight) in *o*-dichlorobenzene (DCB), at a concentration of 10 mg mL^−1^ for polymer. Then, the cathode (the calcium layer about 20 nm, the aluminum layer about 100 nm) was deposited on the polymer layer. The thickness of the active layer was ca. 100 nm, measured with an Ambios Tech XP-2 profilmeter. The effective area of one cell was ca. 4 mm^2^. The *J*-*V* curve was measured using a Keithley 236 source measure unit. Photocurrent was measured with a solar simulator with an AM 1.5 filter at 100 mW cm^−2^ was used as the white-light source. 

### 2.3. Synthesis of the Polymers

The synthesis of the polymers was carried out using palladium-catalyzed Stille-coupling between monomer 4,7-bis(5-bromothiophen-2-yl)-2-(2-ethylhexyl)-2*H*-benzo[d][1,2,3]-triazole derivatives (1 eq) and DTPa-distannyl (1.02 eq), as shown in Scheme 2. Monomers were dissolved in toluene, then Pd_2_(dba)_3_ (2.5% in mol) and (*o*-tol)_3_P (20% in mol) were added to the reaction vial with a magnetic stirring bar. After being purged by three freeze–pump–thaw cycles, the mixture was heated for 24 h at 100 °C. Then, to the reaction mixture, bromobenzene (5 eq) was added as end-cappers and stirred for another 6 h. After the reaction was cooled to room temperature, the reaction mixture was added dropwise to 120 mL of ethanol. The precipitate was extracted successively by methanol, hexane, chloroform, and chlorobenzene using a Soxhlet extractor. Then, chromatography was performed to further purify the polymers. After the standard operation, the products were dried in a vacuum oven overnight to yield the titled polymer. 

**PDTPa-TBTA.** Yield: 68%; *M*_n_ = 24.6 kDa, *M*_w_ = 56.6 kDa, PDI = 2.3; Anal. Calcd for (C_55_H_74_N_2_OS_5_)_n_: C, 70.70; H, 7.79; N, 4.85. Found: C, 70.86; H, 7.89; N, 4.78.

**PDTPa-TffBTA.** Yield: 66%; *M*_n_ = 28.4 kDa, *M*_w_ = 51.1 kDa, PDI = 1.8; Anal. Calcd for (C_55_H_73_FN_2_OS_5_)_n_: C, 67.88; H, 7.26; N, 4.66. Found: C, 68.01; H, 7.38; N, 4.58.

## 3. Results and Discussion

### 3.1. Optical and Electronic Properties

**Figure 1:**
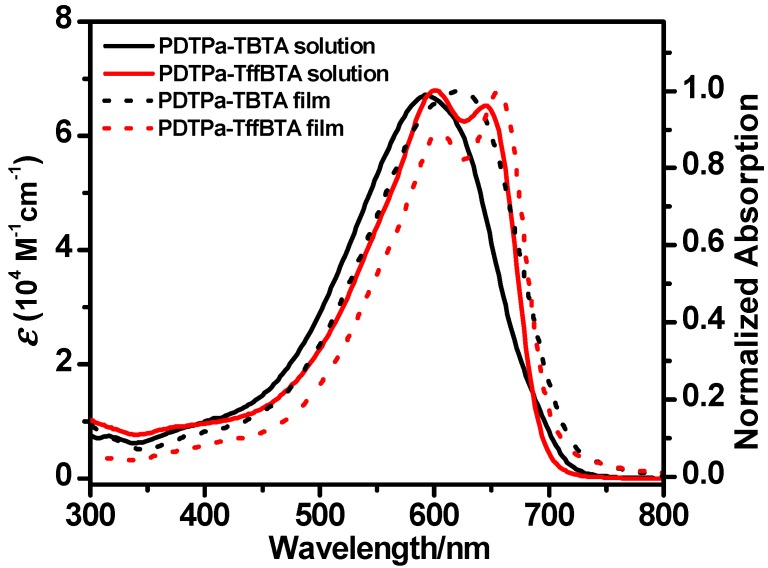
Absorption spectra of **PDTPa-TBTA** and **PDTPa-TffBTA** in a dilute chloroform solution and as thin films.

Absorption spectra of the polymer **PDTPa-TBTA** and **PDTPa-TffBTA** in a dilute chloroform solution and as spin-coated thin films are shown in Figure 1. The detailed absorption data, including the absorption maxima (*λ*_abs_) in solution and film, as well as the corresponding absorption edge (onset, *λ*_onset_) and band gaps (*E*_g_^opt^), are presented in Table 1.

Both of the absorption spectra recorded from dilute chloroform solutions feature a single absorption peak. The molar extinction coefficient (*ε*) of the polymer **PDTPa-TBTA** is 6.7 × 10^4^ M^−1^ cm^−1^ in chloroform. Compared to that of **PDTPa-TBTA** in solution, the absorption spectrum in film is redshifted in the absorption maximum, from 593 to 622 nm. While the onset wavelength of the absorption spectra locate at around 715 nm, almost the same in solution and film, corresponding to a medium bandgap of 1.73 eV. 

The fluorinated polymer **PDTPa-TffBTA** show clear signs of aggregation even in solution, with an absorption peak at 601 nm and a well-marked vibronic shoulder at 645 nm. The fluorination slightly improves the molar extinction coefficient of **PDTPa-TffBTA** to 6.8 × 10^4^ M^−1^ cm^−1^. In a solid film, the shoulder peak is enhanced to be the absorption maximum, indicating strong intermolecular interactions existing in the solid state. At the same time, the absorption peaks bathochromic shift in film, from 601 and 645 nm to 604 and 656 nm, respectively, compared with those in the solution. The onset wavelength is redshifted from 692 to 700 nm, corresponding to a bandgap of 1.79 and 1.77 eV. In addition, the introduction of fluorine in benzotriazole causes a redshift of the absorption maximum and a blueshift in the absorption edge (onset wavelength) of the absorption spectra in both the solution and solid films.

**Figure 2:**
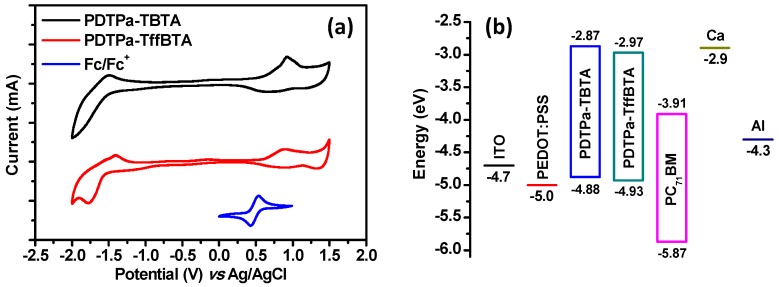
(**a**) Cyclic voltammograms of the polymer films on Pt electrode in 0.1 mol L^−1^ Bu_4_NPF_6_, CH_3_CN solution with a scan rate of 100 mV s^−1^. (**b**) Energy levels with respect to the vacuum of the materials used in the photovoltaic cells.

Energy levels of the highest occupied molecular orbital (HOMO), the lowest unoccupied molecular orbital (LUMO) energy levels (vs. vacuum) of the polymers were determined from the onset oxidation (*E*_OX_) and reduction potentials (*E*_red_) in the cycle voltammogram, which correspond to the ionization potential (IP) and electron affinity (EA), respectively. Cycle voltammograms of the polymer films and standard substance ferrocene are shown in Figure 2a. The HOMO and LUMO energy levels and electrochemical band gaps (*E*_g_^CV^) of the polymers are calculated and summarized in Table 2. The onset oxidation and reduction potential of **PDTPa-TBTA** are 0.56 and −1.45 V vs. Ag/AgCl, corresponding to HOMO and LUMO energy levels of −4.88 and −2.87 eV, respectively. The introduction of fluorine atoms on the **BTA** unit slightly decreases the HOMO energy levels of the polymer **PDTPa-TffBTA** to −4.93 eV. While the LUMO energy level of **PDTPa-TffBTA** is decreased to −2.97eV. The lower HOMO levels of **PDTPa-TffBTA** should result in better oxidative stability in ambient conditions and be propitious to achieve a higher open-circuit voltage (*V*_oc_) in photovoltaic devices.

### 3.2. Photovoltaic Properties

**Figure 3:**
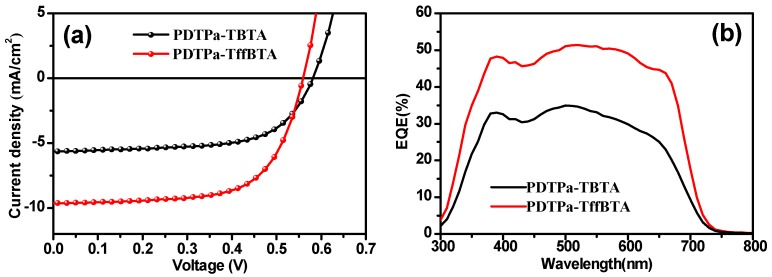
Performance of PSCs under simulated 100 mW cm^−2^ AM1.5G illumination. *J*–*V* curves (**a**) and EQE spectra (**b**) of the devices ITO/PEDOT:PSS/**PDTPa-TBTA**: PC_71_BM (1:1.5, *w/w*) or **PDTPa-TffBTA**: PC_71_BM (1:2, *w/w*)/Ca (20 nm)/Al (100 nm).

To investigate the effect of fluorination on the photovoltaic performance of the asymmetric-building-block-based polymers, bulk heterojunction polymer solar cells were fabricated. Energy levels with respect to the vacuum of the materials used in the photovoltaic cells are shown in Figure 2b. Figure 3a shows the current density (*J*)-potential (*V*) characteristic of PSCs based on the blends of polymer:PC_71_BM under 100 mW cm^−2^ illumination (AM1.5G). The open-circuit voltage (*V*_oc_), the short-circuit current (*J*_sc_), the fill factor (FF), the power conversion efficiency (PCE), and the hole mobility (*μ*_h_) of the PSCs are summarized in Table 3.

In most cases, the introduction of the electron-withdrawing fluorine atom on polymers will lower HOMO energy levels of the resulting polymers and consequently increase the *V*_oc_ of the corresponding solar cells [30,31,32]. Surprisingly, it is found that, with the substitution of fluorine atom on the **BTA** unit, the *V*_oc_ of the devices decreased from 0.58 V for **PDTPa-TBTA** to 0.52 V for **PDTPa-TffBTA**-based PSCs, although the HOMO energy level indeed decreased. A similar abnormal phenomenon has been also found in several benzothiadiazole-based polymer solar cells [33,34,35,36,37,38], and the discrepancy was explained in terms of the morphology of the blended active layer. The **PDTPa-TBTA** gives a PCE of 2.22%, with a *J*_sc_ of 6.04 mA cm^−2^ and an FF of 0.63 without thermal annealing treatment. For the fluorine substituted polymer, **PDTPa-TffBTA** showed higher PCE of 3.43%, an FF of 0.64 and a significantly increased *J*_sc_ of 10.23 mA cm^−2^. 

**Figure 4:**
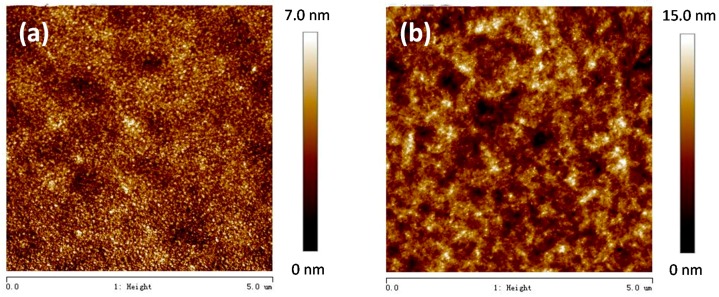
AFM height image of (**a**) **PDTPa-TBTA**:PC_71_BM (1:1.5, *w/w*) and (**b**) **PDTPa-TffBTA**: PC_71_BM (1:2, *w/w*) active layers.

In order to investigate the effect of fluorination of the polymers on the morphologies and microstructures of the active layers of the PSCs, tapping-mode atom force microscopy (AFM) was employed to analyze active layers of the polymer solar cells. As shown in Figure 4, **PDTPa-TBTA**:PC_71_BM film displayed very smooth and uniform surface with a root-mean-square (RMS) of 0.99 nm. The introduction of fluorine on **BTA** made the phase separate clearly in the **PDTPa-TffBTA**:PC_71_BM blend with an increased RMS of 2.14 nm, indicating an increase of aggregation domain size in the blend. The enlargement in the aggregation domain size of the active layer could be beneficial to the charge carrier transport in the bulk heterojunction (BHJ) active layers. The law is in accordance with the effect of fluorination of most photovoltaic polymers in previous reports [16,17,29,30,31]. However, the morphology of the active layers was inadequate to explain the discrepancy in the *V*_oc_ of solar cells based on the **DTPa-BTA** copolymers. As discussed in our previous work, there might be additional energy loss (*V*_oc_ loss) in some BHJ photovoltaic materials system [39]. Due to the complexity of influencing factors of *V*_oc_ [40], the reason for the discrepancy that fluorination of the donor polymer decreased open-circuit voltage of corresponding solar cells is still unclear and needs further study.

The photovoltaic properties of the polymers could be further investigated by the external quantum efficiency (EQE) spectra of the devices. Both polymers show broad photo response from 300 to nearly 750 nm, and the flat EQE values, as shown in Figure 3b, indicate the balanced contribution from the polymers and acceptor PC_71_BM in both of **PDTPa-BTA**- and **PDTPa-ffBTA**-based devices. The significant increase in the EQE value proves more efficient photon harvesting and charge collection in the **PDTPa-TffBTA**-based active layers, than that of **PDTPa-TBTA**.

**Figure 5:**
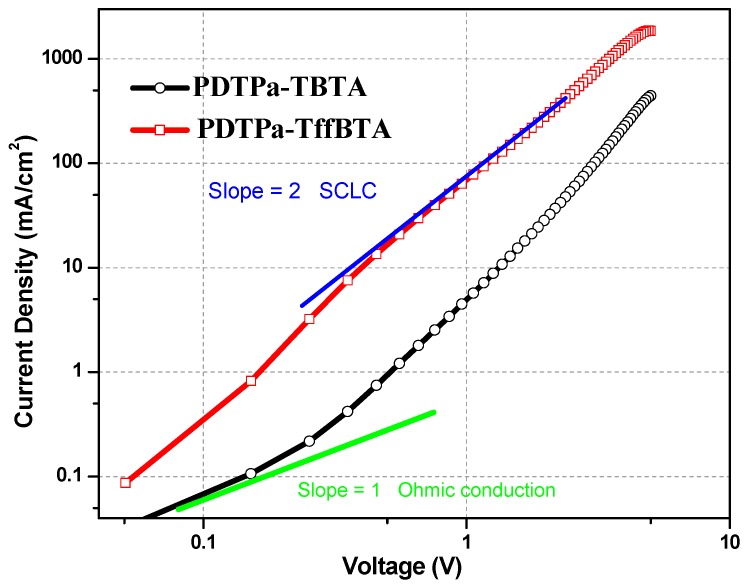
Typical *J*-*V* curves based on the hole-only devices of ITO/PEDOT:PSS/active layer/Au.

To further understand the difference device performance between **PDTPa-TBTA** and **PDTPa-TffBTA**, the charge transport properties of the active layers (polymer:PC_71_BM blends) were investigated using the space charge limited current (SCLC) method with the hole only device with a configuration of ITO/PEDOT:PSS/polymer:PC_71_BM/Au. The experimental dark current densities of the polymer:PC_71_BM blends were measured when applied with voltage from −5 to 5 V. The applied voltage was corrected from the voltage drop *V*_rs_ due to the series resistance and contact resistance from ITO/PEDOT:PSS, which were measured to be about 35 Ω from a reference device without the layer of polymer:PC_71_BM. From the plots of *J*^0.5^ vs. *V*, the hole mobilities of the polymers were deduced from the Mott–Gurneys law [41]:

$$J=\frac{9}{8}\varepsilon_{r}\varepsilon_{0}\mu_{h}\frac{V^{2}}{L^{3}}$$

where *ε*_r_ is the dielectric constant of the polymer, which is assumed to be around 3, *ε*_0_ is the permittivity of free space, *μ*_h_ is the hole mobility, *V* is the voltage drop across the device, and *L* is the film thickness of the active layer. The plots of the current density vs. voltage for the devices are shown in Figure 5. The hole mobility of **PDTPa-TffBTA**/PC_71_BM film are calculated to be 2.66 × 10^−4^ cm^2^ v^−1^ s^−1^, which is much higher than that of **PDTPa-TBTA**/PC_71_BM of 1.61 × 10^−5^ cm^2^ v^−1^ s^−1^. Apparently, charge carrier mobilities were increased for extent and which is one important reason for their *J*_sc_ and high PCE values. Besides the optical properties, the increase in charge carrier mobilities should be another important reason for the obvious promotion of *J*_sc_ and PCE values in **PDTPa-TffBTA**-based solar cells, compared to those of **PDTPa-TBTA**.

## 4. Conclusions

In summary, two new conjugated polymers with 5*H*-dithieno[3,2-b:2′,3′-d]pyran (**DTPa**) as an electron donor and benzo[d][1,2,3]triazole (**BTA**) or di-fluorinated benzo[d][1,2,3] triazole (**ffBTA**) as an electron acceptor with thiophene as a π-bridge were synthesized and characterized. To the best of our knowledge, it is the first time to copolymerize **BTA** with asymmetric electron-donating building blocks. Two asymmetric-building-block-containing polymers (ABC-polymers) possess strong and broad absorption in the range of 300–750 nm and a medium optical bandgap of 1.73 and 1.77 eV for **PDTPa-TBTA** and **PDTPa-TffBTA**, respectively. With the introduction of fluorine on the **BTA** unit, the absorption peaks redshift and obvious shoulder peak appear, indicating strong intermolecular interactions existing in the fluorinated polymer system. Interestingly, although the HOMO energy level indeed decreased, the fluorination decreased the *V*_oc_ of ABC polymers from 0.58 V for **PDTPa-TBTA** to 0.52 V for **PDTPa-TffBTA**. At the same time, the introduction of fluorine on the **BTA** unit remarkably improved *J*_sc_ of the solar cells based on the resulting polymer by nearly 70%. Together with an outstanding FF of 0.64, fluorination increased the PCE of the solar cells by nearly 55%, from 2.22% for that of **PDTPa-TBTA** to 3.43% for that of **PDTPa-TffBTA**. These results indicate that the introduction of fluorine on the **BTA** unit is also effective in improving the photovoltaic performance of ABC polymers. 

## Figures and Tables

**Table 1:** Optical properties and experimental and calculated band gaps of the **DTPa**-based polymers.

Polymer	*λ*_abs_ in CHCl_3_ (nm)	*λ*_abs_ in Films (nm)	*λ*_onset_ in Films (nm)	*E*_g_^opt^ (eV)
**PDTPa-TBTA**	593	622	715	1.73
**PDTPa-TffBTA**	601, 645	604, 656	700	1.77

**Table 2:** Electrochemical properties of the **DTPa**-based polymers.

Polymer	*E*_OX_ (V)	HOMO (eV)	*E*_red_ (V)	LUMO (eV)	*E*_g_^CV^ (eV)
**PDTPa-TBTA**	0.56	−4.88	−1.45	−2.87	1.96
**PDTPa-TffBTA**	0.61	−4.93	−1.55	−2.97	2.01

**Table 3:** Photovoltaic performance of PSCs with the device structure of ITO/PEDOT:PSS/**PDTPa-TBTA**: PC_71_BM (1:1.5, *w/w*) or **PDTPa-TffBTA**: PC_71_BM (1:2, *w/w*)/Ca (20 nm)/Ag (100 nm).

Polymer	*V*_oc_ [v]	*J*_sc_ [mA cm^−2^]	FF	PCE_max_ (PCE_ave_^a^) [%]	Hole Mobility ^b^ [cm^2^ v^−1^ s^−1^]
**PDTPa-TBTA**	0.58	6.04	0.63	2.22 (2.17)	1.61 × 10^−5^
**PDTPa-TffBTA**	0.52	10.23	0.64	3.43 (3.20)	2.66 × 10^−4^

^a^ The average PCE is obtained from over 10 devices. ^b^ Measured via a space charge limited current (SCLC) method.
